# Solitary fibrous tumor of the pleura presenting with syncope episodes when coughing

**DOI:** 10.1186/1477-7819-6-86

**Published:** 2008-08-19

**Authors:** Luigi Santambrogio, Mario Nosotti, Alessandro Palleschi, Lorenzo Rosso, Davide Tosi, Matilde De Simone, Michele M Ciulla, Marco Maggioni, Ugo Cioffi

**Affiliations:** 1 Department of Surgery, Thoracic Unit, Fondazione Ospedale Maggiore Policlinico, Mangiagalli e Regina Elena, IRCCS, Milan, Italy; 2 Department of Surgery, Fondazione Ospedale Maggiore Policlinico, Mangiagalli e Regina Elena, IRCCS, Milan, Italy; 3 Istituto di Medicina Cardiovascolare, Centro di Fisiologia Clinica e Ipertensione, University of Milan, Fondazione Ospedale Maggiore Policlinico, Mangiagalli e Regina Elena, IRCCS, Milan, Italy; 4 A.O. San Paolo, U.O. Anatomia Patologica, Milan, Italy

## Abstract

**Background:**

Solitary fibrous tumor of the pleura is a rarely encountered clinical entity which may have different clinical pictures. Although the majority of these neoplasms have a benign course, the malignant form has also been reported.

**Case presentation:**

We herein describe a case of 72 year-old man with head, facial, and thoracic traumas caused by neurally-mediated situational syncope when coughing. The diagnostic work-up including chest x-ray, CT and PET, revealed a large solitary mass of the left hemithorax. Radical surgical resection of the mass was performed through a left lateral thoracotomy and completed with a wedge resection of the lingula. Hystological examination of the surgical specimen showed an encapsulated mass measuring 12 × 11.5 × 6 cm consistent with a solitary fibrous tumor of the pleura. It's surgical removal definitively resolved the neurologic manifestations. The patient had no postoperative complications. At two years follow-up the patient is free from recurrence and without clinical manifestations.

**Conclusion:**

In our case its resection definitively resolved the episodes of situational syncope due, in our opinion, to the large thoracic mass compressing the phrenic nerve

## Background

First described by Klemperer and Rabin [1], the solitary fibrous tumor of the pleura (SFTP) is a localized benign neoplasm arising from the submesothelial mesenchymal layer [2] even if malignant forms have also been described [3]. With about 800 cases reported in the world literature, this rare entity contrast with the primary diffuse pleural mesothelioma that have an incidence of 3000 new cases every year in the USA [4]. In over half of patients the tumor is asymptomatic, but if symptoms occur then chest pain, cough and dyspnea are the most common complaints. Complete en bloc surgical resection is the treatment of choice for these neoplasms offering a cure in all patients with benign form even if tumor recurrence may occur also in tumors with benign histological features [4,5]. We describe an unreported case of SFTP, to our knowledge, manifesting with syncope episodes when coughing.

## Case presentation

A 72 year-old man was admitted to the hospital for head injury, facial and left hemithorax contusions. The patient referred to had fainted after coughing; the same symptoms had occurred six and three months earlier. Diverticulosis of the colon was the only disease reported by the patient in his medical history. He denied smoking, drug or alcohol abuse. Physical examination showed dullness to percussion and decreased breath sound in the affected hemithorax. The neurological examination was negative. Blood pressure was 170/80 mmHg, heart rate was 90 beats/minute and rhythmic. Laboratory findings, arterial gas analysis, electrocardiogram, and brain computed tomography were negative.

**Figure 1:**
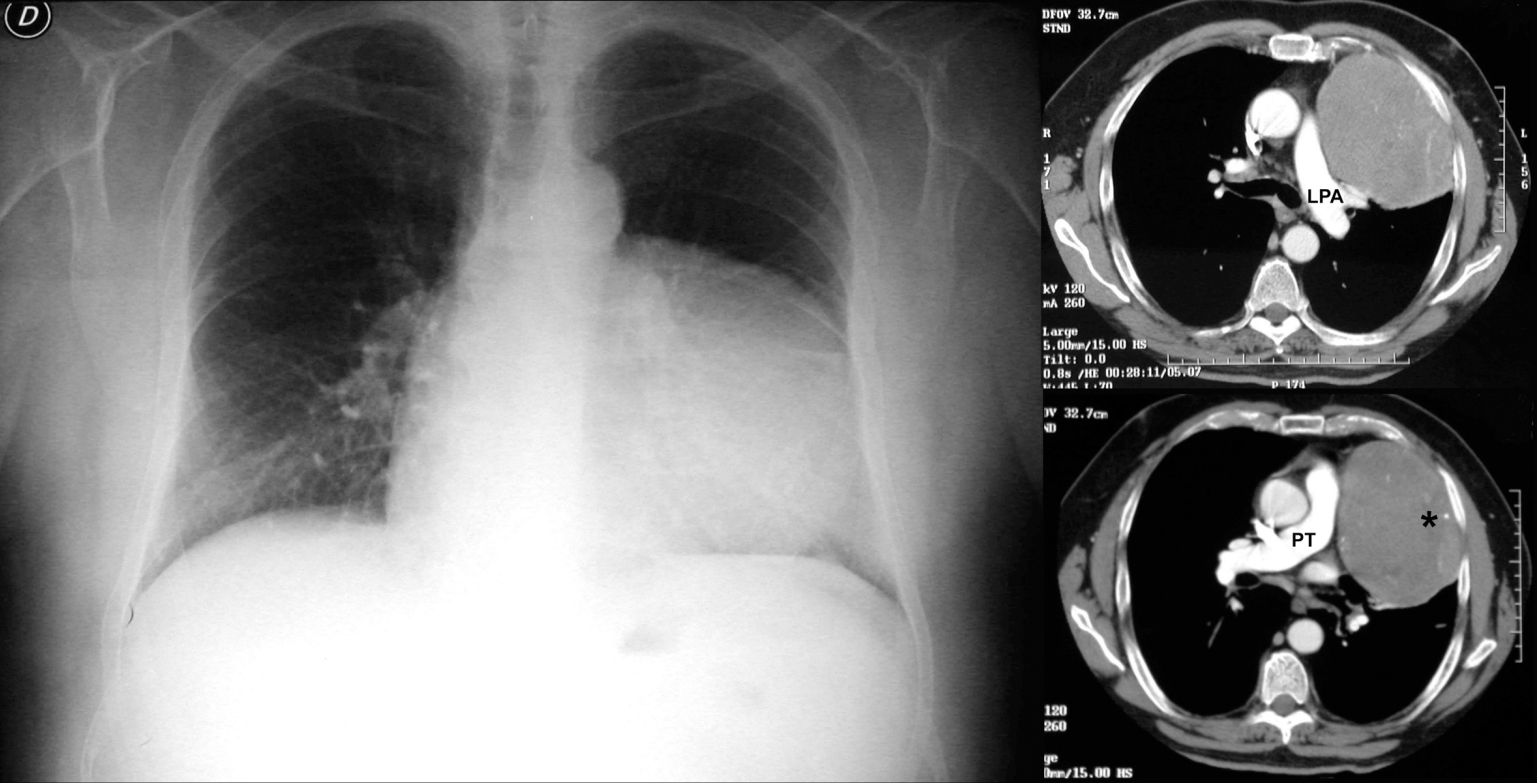
*Left panel*: chest x-ray showing a large opacity on the left side. *Right panel*: the CT scan of the chest showing a solid mass of 11 × 8 cm in the left hemi thorax, with vertical extension of 10 cm, mild enhancement after contrast medium infusion and some calcifications in the basal part (asterisk). The lesion is in close relation with chest wall, left pulmonary artery (LPA) and pulmonary trunk (PT), without signs of local infiltration.

**Figure 2:**
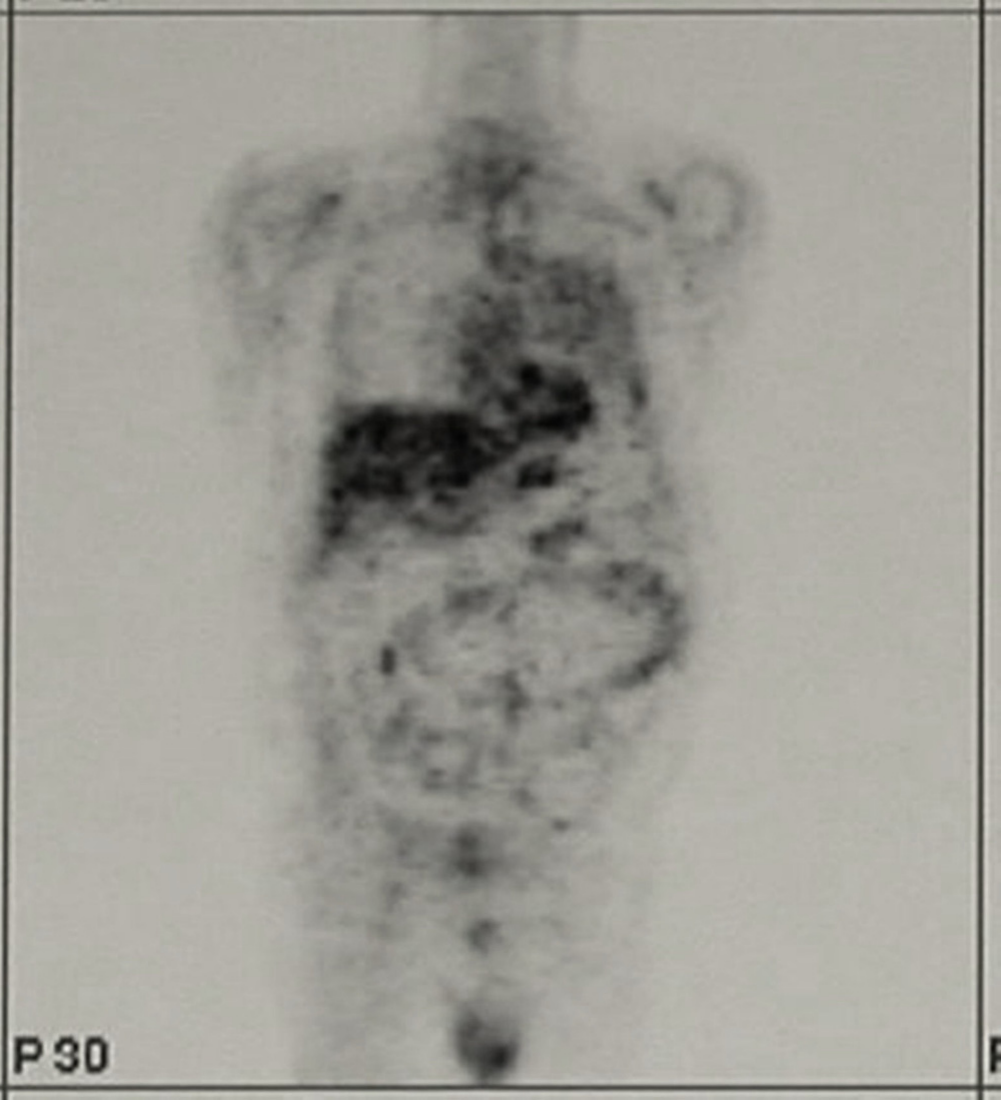
Moderate activity of the mass on Positron Emission Tomography study.

A chest x-ray revealed fractures of three left ribs plus a large medium-basal opacity on the left hemithorax (Figure 1a). Computed tomography (CT) of the thorax confirmed the presence of a well-delineated, homogeneous, solid mass of 11 × 8 cm, extending for about 10 cm on the vertical axis. The mass presented a mild enhancement after contrast injection and calcifications in the basal part. It was close to the chest wall, adjacent to the left pulmonary artery, pulmonary artery trunk, and left ventricle with no signs of infiltration (Figure 1b). Bronchoscopy showed an insignificant bleeding from the upper left bronchus. Positron emission tomography (PET) revealed a mild positivity of the lesion (Figure 2). Echocardiogram, Holter ECG monitoring, and carotid Doppler ultrasonography were negative. With suspected diagnosis of SFTP, the patient underwent surgery. Through a left lateral thoracotomy, the neoplasm was carefully isolated, and its origin from the visceral pleura of the pulmonary lingula segment became evident. The adhesions with the phrenic nerve were cut preserving the nerve integrity. The mass excision was performed with clear surgical margins and completed with a wedge resection of the lingula. The postoperative course was uneventful, a good re-expansion of the left lung was obtained, and the patient was discharged on the fifth postoperative day.

**Figure 3:**
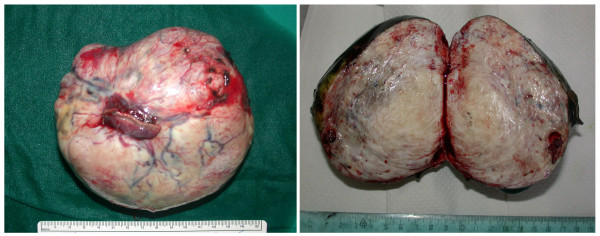
*Left panel*: surgical specimen with detail of the wedge resection of the lingula. *Right panel*: solitary fibrous tumor of the pleura, whorled fibrous tissue is evident on the cut section.

**Figure 4:**
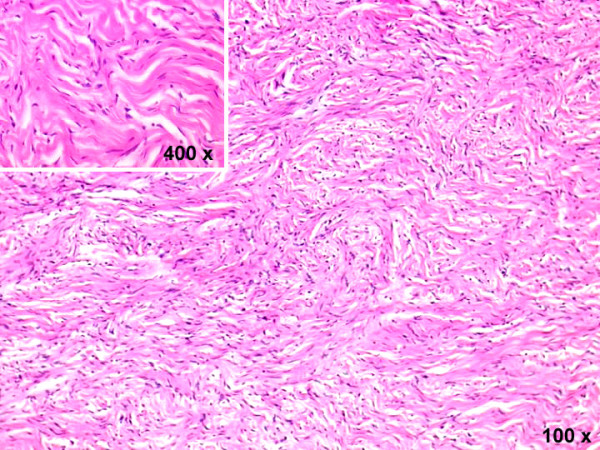
Tumor consists of elongated cells that display a storiform pattern of growth and abundant stromal collagen (hematoxylin & eosin stain; magnification 100 ×). At higher magnification, tumor cells appear of small size, spindle, with no cytologic atypia (insert; magnification 400 ×).

Pathological examination showed a 12 × 11, 5 × 6 cm encapsulated tumor mass (Figure 3a), whitish in color, with whorled appearance and calcification on cut section (Fig. 3b). Microscopic examination showed fibroblast-like structures within the collagen (Figure 4). The diagnosis of benign SFTP was confirmed by mmunohistochemical analysis (CD34+, BCL-2+, SMA-, S100-).

After two years of follow-up the patient is in good clinical condition without recurrence of disease or clinical symptoms.

## Discussion

It is well known that solitary fibrous tumors of the pleura are incidentally discovered during chest x-ray examination because these neoplasms often have a silent clinical course for several years [4,6]. It has been described in all ages, but the peak of incidence is in the sixth and seventh decades of life [1]. The larger the tumor, the more likely it is that there will be symptoms [2,4]. Systemic symptoms such as weight loss, nocturnal sweating, chills, weakness, digital clubbing, hypertrophic osteoarthropathy, and hypoglycemia have also been reported [6,7]. Hypertrophic osteoarthropathy (Pierre Marie-Bamberg syndrome) [6-8], is related to the abnormal production of hyaluronic acid by tumor cells and affect up to 20% of patients. In less than 5% of cases, SFTP can secrete insulin-like growth factor II which causes refractory hypoglycemia [1,7]. Sometimes, large tumors might present an unusual onset, such as the case of Shaker and *et al*, [8] in which a woman with leg edema and dyspnea caused by a large SFTP compressing the right atrium and the inferior vena cava is described. In our case, the large tumor presented with episodes of situational syncope when coughing. Situational syncope is a neurally-mediated syncope related to a reflex response that, when triggered, determines vasodilatation and bradycardia. Neurally-mediated syncope is usually classified as vasovagal (common faint), or situational [9]. Suggestive for vasovagal syncope are a long history of syncope, a youthful age, a sudden and unpleasant sight, pain or sound, prolonged standing in hot and/or crowded places. It often associates with nausea and vomiting. Situational syncope is diagnosed if syncope occurs during or after urination, defecation, cough or swallowing [9]. In our case the syncope occurred immediately after coughing, without nausea and vomiting; in addition, the patient was old and reported a trauma. We, therefore, hypothesize that coughing, due to the stimulation of the phrenic nerve, resulted in a high intrathoracic pressure producing an exaggerated Valsalva responce that decreased venous return and, consequently, cardiac output. At this regard it should be noticed that the accidental phrenic nerve injury produces cough and dyspnea and this evenience is well documented during right atrial catheterization procedures [10]. All these details ruled out the possibility of a common faint, and consequently a diagnosis of situational syncope when coughing was made. The negative results of the cardiovascular tests associated to the presence of a large thoracic mass convinced us to consider the cough syncope related to the stimulation of the phrenic nerve by the neoplasm. In fact, after surgical removal of the tumor, the patient is free from syncope episodes confirming the direct implication of the solitary fibrous tumor of the pleura in the neurologic manifestations.

## Conclusion

Generally SFTP is a localized, benign tumor which may have different clinical pictures. It is curable using a careful and complete resection, provided that the surgical margins are free from neoplastic cells. In our case its resection definitively resolved the episodes of situational syncope due, in our opinion, to the large thoracic mass compressing the phrenic nerve.

## Consent

Written and informed consent was obtained from the patient for publication of this case report and any accompanying images. A copy of the written consent is available for review by the Editor-in-Chief of this journal.

## Competing interests

The authors declare that they have no competing interests.

## Authors' contributions

LS conceived the idea, did supervision of manuscript preparation and proof reading initiated treatment, did surgical procedures and approved the final version of the paper. MN, AP, LR, DT proof reading, initiated treatment, did surgical procedures. UC, MDS wrote the manuscript and carried out literature review; MMC contributed to data management and preparing of the manuscript. All authors read and approved the final manuscript.
